# Supplementary material for: Genome-Wide Identification and Expression Analysis of DWARF53 Gene in Response to GA and SL Related to Plant Height in Banana
Source: Plants (Basel). 2024 Feb 5;13(3):458. doi: 10.3390/plants13030458 (PMC10857657; doi:10.3390/plants13030458)
Supplement: Supplementary file 1 [file plants-13-00458-s001.zip › plants-2811594-supplementary.pdf]

Genome-Wide Identification and Expression Analysis of *DWARF53* Gene in Response to GA and SL Related to Plant Height in Banana

Ning Tong, Chunyu Zhang, Xiaoqiong Xu, Zhilin Zhang, Jiahui Li, Zhaoyang Liu, Yukun Chen, Zihao Zhang, Yuji Huang, Yuling Lin and Zhongxiong Lai *

Institute of Horticultural Biotechnology, Fujian Agriculture and Forestry University, Fuzhou, 350002, China

* Correspondence: E-mail address: laizx01@163.com

Table S1: Similarity of D53 protein sequences.

Gene name	species name	Gene name	Species name	Similarity/%
MaD53-1	*M. acuminata*	MaD53-2	*M. acuminata*	72.449
MaD53-1	*M. acuminata*	MaD53-3	*M. acuminata*	64.7364
MaD53-1	*M. acuminata*	MbD53-1	*M. balbisiana*	97.1477
MaD53-1	*M. acuminata*	MbD53-2	*M. balbisiana*	71.912
MaD53-1	*M. acuminata*	MbD53-3	*M. balbisiana*	72.5779
MaD53-1	*M. acuminata*	MiD53-1	*M. itinerans*	83.4443
MaD53-1	*M. acuminata*	MiD53-2	*M. itinerans*	57.4788
MaD53-1	*M. acuminata*	LZmD53	*Zostera marina*	27.4755
MaD53-1	*M. acuminata*	PeD53L	*Phalaenopsis equestris*	33.7148
MaD53-1	*M. acuminata*	DcD53	*Dendrobium catenatum*	39.3862
MaD53-1	*M. acuminata*	TaD53-2	*Triticum aestivum*	28.6351
MaD53-1	*M. acuminata*	TaD53-1	*Triticum aestivum*	29.1332
MaD53-1	*M. acuminata*	TaD53-3	*Triticum aestivum*	32.2954
MaD53-1	*M. acuminata*	TaD53-4	*Triticum aestivum*	27.9008
MaD53-1	*M. acuminata*	TaD53-5	*Triticum aestivum*	33.156
MaD53-1	*M. acuminata*	TaD53L	*Triticum aestivum*	28.9028
MaD53-1	*M. acuminata*	TuD53L	*Triticum urartu*	33.0674
MaD53-1	*M. acuminata*	TdD53-1	*Triticum dicoccoides*	29.1332
MaD53-1	*M. acuminata*	TdD53-2	*Triticum dicoccoides*	29.7056
MaD53-1	*M. acuminata*	TdD53-3	*Triticum dicoccoides*	33.156
MaD53-1	*M. acuminata*	TdD53-4	*Triticum dicoccoides*	27.9008
MaD53-1	*M. acuminata*	PhD53	*Panicum hallii*	31.7376
MaD53-1	*M. acuminata*	ZmD53	*Zea mays*	25.7269
MaD53-1	*M. acuminata*	SbD53-1	*Sorghum bicolor*	30.2305
MaD53-1	*M. acuminata*	SbD53-2	*Sorghum bicolor*	23.155
MaD53-1	*M. acuminata*	OsD53	*Oryza sativa Japonica Group*	30.6808
MaD53-1	*M. acuminata*	OsD53L	*Oryza sativa Japonica Group*	30.4694
MaD53-1	*M. acuminata*	ObD53	*Oryza brachyantha*	25.0223
MaD53-1	*M. acuminata*	ObD53L	*Oryza brachyantha*	26.7618
MaD53-2	*M. acuminata*	MaD53-3	*M. acuminata*	57.5627
MaD53-2	*M. acuminata*	MbD53-1	*M. balbisiana*	72.449

MaD53-2	*M. acuminata*	MbD53-2	*M. balbisiana*	96.8537
MaD53-2	*M. acuminata*	MbD53-3	*M. balbisiana*	58.045
MaD53-2	*M. acuminata*	MiD53-1	*M. itinerans*	57.5642
MaD53-2	*M. acuminata*	MiD53-2	*M. itinerans*	96.7074
MaD53-2	*M. acuminata*	LZmD53	*Zostera marina*	28.0999
MaD53-2	*M. acuminata*	PeD53L	*Phalaenopsis equestris*	34.243
MaD53-2	*M. acuminata*	DcD53	*Dendrobium catenatum*	37.0844
MaD53-2	*M. acuminata*	TaD53-2	*Triticum aestivum*	24.264
MaD53-2	*M. acuminata*	TaD53-1	*Triticum aestivum*	28.9544
MaD53-2	*M. acuminata*	TaD53-3	*Triticum aestivum*	32.3843
MaD53-2	*M. acuminata*	TaD53-4	*Triticum aestivum*	32.2409
MaD53-2	*M. acuminata*	TaD53-5	*Triticum aestivum*	32.1809
MaD53-2	*M. acuminata*	TaD53L	*Triticum aestivum*	24.1748
MaD53-2	*M. acuminata*	TuD53L	*Triticum urartu*	32.2695
MaD53-2	*M. acuminata*	TdD53-1	*Triticum dicoccoides*	28.9544
MaD53-2	*M. acuminata*	TdD53-2	*Triticum dicoccoides*	23.9072
MaD53-2	*M. acuminata*	TdD53-3	*Triticum dicoccoides*	32.1809
MaD53-2	*M. acuminata*	TdD53-4	*Triticum dicoccoides*	32.3295
MaD53-2	*M. acuminata*	PhD53	*Panicum hallii*	29.344
MaD53-2	*M. acuminata*	ZmD53	*Zea mays*	35.6828
MaD53-2	*M. acuminata*	SbD53-1	*Sorghum bicolor*	27.8369
MaD53-2	*M. acuminata*	SbD53-2	*Sorghum bicolor*	22.8782
MaD53-2	*M. acuminata*	OsD53	*Oryza sativa Japonica Group*	30.504
MaD53-2	*M. acuminata*	OsD53L	*Oryza sativa Japonica Group*	35.8725
MaD53-2	*M. acuminata*	ObD53	*Oryza brachyantha*	29.5637
MaD53-2	*M. acuminata*	ObD53L	*Oryza brachyantha*	27.1186
MaD53-3	*M. acuminata*	MbD53-1	*M. balbisiana*	71.5644
MaD53-3	*M. acuminata*	MbD53-2	*M. balbisiana*	57.7355
MaD53-3	*M. acuminata*	MbD53-3	*M. balbisiana*	95.5017
MaD53-3	*M. acuminata*	MiD53-1	*M. itinerans*	57.3739
MaD53-3	*M. acuminata*	MiD53-2	*M. itinerans*	49.7648
MaD53-3	*M. acuminata*	LZmD53	*Zostera marina*	27.4755
MaD53-3	*M. acuminata*	PeD53L	*Phalaenopsis equestris*	38.9965
MaD53-3	*M. acuminata*	DcD53	*Dendrobium catenatum*	34.3993
MaD53-3	*M. acuminata*	TaD53-2	*Triticum aestivum*	26.5834
MaD53-3	*M. acuminata*	TaD53-1	*Triticum aestivum*	27.6139
MaD53-3	*M. acuminata*	TaD53-3	*Triticum aestivum*	33.363
MaD53-3	*M. acuminata*	TaD53-4	*Triticum aestivum*	28.7865
MaD53-3	*M. acuminata*	TaD53-5	*Triticum aestivum*	33.6879
MaD53-3	*M. acuminata*	TaD53L	*Triticum aestivum*	22.2123
MaD53-3	*M. acuminata*	TuD53L	*Triticum urartu*	33.5993
MaD53-3	*M. acuminata*	TdD53-1	*Triticum dicoccoides*	27.6139
MaD53-3	*M. acuminata*	TdD53-2	*Triticum dicoccoides*	28.5459
MaD53-3	*M. acuminata*	TdD53-3	*Triticum dicoccoides*	33.6879

MaD53-3	*M. acuminata*	TdD53-4	*Triticum dicoccoides*	26.4836
MaD53-3	*M. acuminata*	PhD53	*Panicum hallii*	28.1915
MaD53-3	*M. acuminata*	ZmD53	*Zea mays*	27.8414
MaD53-3	*M. acuminata*	SbD53-1	*Sorghum bicolor*	30.1418
MaD53-3	*M. acuminata*	SbD53-2	*Sorghum bicolor*	27.214
MaD53-3	*M. acuminata*	OsD53	*Oryza sativa Japonica Group*	28.1167
MaD53-3	*M. acuminata*	OsD53L	*Oryza sativa Japonica Group*	29.6723
MaD53-3	*M. acuminata*	ObD53	*Oryza brachyantha*	27.3375
MaD53-3	*M. acuminata*	ObD53L	*Oryza brachyantha*	22.7475
MbD53-1	*M. balbisiana*	MbD53-2	*M. balbisiana*	71.912
MbD53-1	*M. balbisiana*	MbD53-3	*M. balbisiana*	69.3772
MbD53-1	*M. balbisiana*	MiD53-1	*M. itinerans*	75.2617
MbD53-1	*M. balbisiana*	MiD53-2	*M. itinerans*	62.9351
MbD53-1	*M. balbisiana*	LZmD53	*Zostera marina*	35.7716
MbD53-1	*M. balbisiana*	PeD53L	*Phalaenopsis equestris*	39.2606
MbD53-1	*M. balbisiana*	DcD53	*Dendrobium catenatum*	41.5175
MbD53-1	*M. balbisiana*	TaD53-2	*Triticum aestivum*	29.6164
MbD53-1	*M. balbisiana*	TaD53-1	*Triticum aestivum*	29.6693
MbD53-1	*M. balbisiana*	TaD53-3	*Triticum aestivum*	32.3843
MbD53-1	*M. balbisiana*	TaD53-4	*Triticum aestivum*	34.3667
MbD53-1	*M. balbisiana*	TaD53-5	*Triticum aestivum*	34.2199
MbD53-1	*M. balbisiana*	TaD53L	*Triticum aestivum*	29.7056
MbD53-1	*M. balbisiana*	TuD53L	*Triticum urartu*	34.1312
MbD53-1	*M. balbisiana*	TdD53-1	*Triticum dicoccoides*	29.6693
MbD53-1	*M. balbisiana*	TdD53-2	*Triticum dicoccoides*	29.7948
MbD53-1	*M. balbisiana*	TdD53-3	*Triticum dicoccoides*	34.2199
MbD53-1	*M. balbisiana*	TdD53-4	*Triticum dicoccoides*	34.4553
MbD53-1	*M. balbisiana*	PhD53	*Panicum hallii*	33.9539
MbD53-1	*M. balbisiana*	ZmD53	*Zea mays*	26.4317
MbD53-1	*M. balbisiana*	SbD53-1	*Sorghum bicolor*	23.1383
MbD53-1	*M. balbisiana*	SbD53-2	*Sorghum bicolor*	26.0148
MbD53-1	*M. balbisiana*	OsD53	*Oryza sativa Japonica Group*	30.8576
MbD53-1	*M. balbisiana*	OsD53L	*Oryza sativa Japonica Group*	30.7352
MbD53-1	*M. balbisiana*	ObD53	*Oryza brachyantha*	28.9403
MbD53-1	*M. balbisiana*	ObD53L	*Oryza brachyantha*	25.1561
MbD53-2	*M. balbisiana*	MbD53-3	*M. balbisiana*	58.1315
MbD53-2	*M. balbisiana*	MiD53-1	*M. itinerans*	57.6594
MbD53-2	*M. balbisiana*	MiD53-2	*M. itinerans*	92.4741
MbD53-2	*M. balbisiana*	LZmD53	*Zostera marina*	27.7431
MbD53-2	*M. balbisiana*	PeD53L	*Phalaenopsis equestris*	38.7324
MbD53-2	*M. balbisiana*	DcD53	*Dendrobium catenatum*	36.3171
MbD53-2	*M. balbisiana*	TaD53-2	*Triticum aestivum*	24.7101
MbD53-2	*M. balbisiana*	TaD53-1	*Triticum aestivum*	31.3673
MbD53-2	*M. balbisiana*	TaD53-3	*Triticum aestivum*	32.0285

MbD53-2	*M. balbisiana*	TaD53-4	*Triticum aestivum*	33.481
MbD53-2	*M. balbisiana*	TaD53-5	*Triticum aestivum*	31.7376
MbD53-2	*M. balbisiana*	TaD53L	*Triticum aestivum*	24.7101
MbD53-2	*M. balbisiana*	TuD53L	*Triticum urartu*	31.8262
MbD53-2	*M. balbisiana*	TdD53-1	*Triticum dicoccoides*	31.3673
MbD53-2	*M. balbisiana*	TdD53-2	*Triticum dicoccoides*	24.7993
MbD53-2	*M. balbisiana*	TdD53-3	*Triticum dicoccoides*	31.7376
MbD53-2	*M. balbisiana*	TdD53-4	*Triticum dicoccoides*	31.9752
MbD53-2	*M. balbisiana*	PhD53	*Panicum hallii*	29.6986
MbD53-2	*M. balbisiana*	ZmD53	*Zea mays*	31.9824
MbD53-2	*M. balbisiana*	SbD53-1	*Sorghum bicolor*	27.7482
MbD53-2	*M. balbisiana*	SbD53-2	*Sorghum bicolor*	23.3395
MbD53-2	*M. balbisiana*	OsD53	*Oryza sativa Japonica Group*	30.8576
MbD53-2	*M. balbisiana*	OsD53L	*Oryza sativa Japonica Group*	36.0496
MbD53-2	*M. balbisiana*	ObD53	*Oryza brachyantha*	29.7418
MbD53-2	*M. balbisiana*	ObD53L	*Oryza brachyantha*	27.2971
MbD53-3	*M. balbisiana*	MiD53-1	*M. itinerans*	57.5642
MbD53-3	*M. balbisiana*	MiD53-2	*M. itinerans*	50.2352
MbD53-3	*M. balbisiana*	LZmD53	*Zostera marina*	27.4755
MbD53-3	*M. balbisiana*	PeD53L	*Phalaenopsis equestris*	36.9718
MbD53-3	*M. balbisiana*	DcD53	*Dendrobium catenatum*	39.7059
MbD53-3	*M. balbisiana*	TaD53-2	*Triticum aestivum*	26.9402
MbD53-3	*M. balbisiana*	TaD53-1	*Triticum aestivum*	24.2181
MbD53-3	*M. balbisiana*	TaD53-3	*Triticum aestivum*	33.274
MbD53-3	*M. balbisiana*	TaD53-4	*Triticum aestivum*	28.1665
MbD53-3	*M. balbisiana*	TaD53-5	*Triticum aestivum*	29.4326
MbD53-3	*M. balbisiana*	TaD53L	*Triticum aestivum*	26.9402
MbD53-3	*M. balbisiana*	TuD53L	*Triticum urartu*	29.344
MbD53-3	*M. balbisiana*	TdD53-1	*Triticum dicoccoides*	24.2181
MbD53-3	*M. balbisiana*	TdD53-2	*Triticum dicoccoides*	27.2079
MbD53-3	*M. balbisiana*	TdD53-3	*Triticum dicoccoides*	29.4326
MbD53-3	*M. balbisiana*	TdD53-4	*Triticum dicoccoides*	28.2551
MbD53-3	*M. balbisiana*	PhD53	*Panicum hallii*	27.1277
MbD53-3	*M. balbisiana*	ZmD53	*Zea mays*	26.0793
MbD53-3	*M. balbisiana*	SbD53-1	*Sorghum bicolor*	30.1418
MbD53-3	*M. balbisiana*	SbD53-2	*Sorghum bicolor*	23.7085
MbD53-3	*M. balbisiana*	OsD53	*Oryza sativa Japonica Group*	28.5588
MbD53-3	*M. balbisiana*	OsD53L	*Oryza sativa Japonica Group*	30.9123
MbD53-3	*M. balbisiana*	ObD53	*Oryza brachyantha*	27.4265
MbD53-3	*M. balbisiana*	ObD53L	*Oryza brachyantha*	25.0669
MiD53-1	*M. itinerans*	MiD53-2	*M. itinerans*	66.5081
MiD53-1	*M. itinerans*	LZmD53	*Zostera marina*	26.9267
MiD53-1	*M. itinerans*	PeD53L	*Phalaenopsis equestris*	36.9172
MiD53-1	*M. itinerans*	DcD53	*Dendrobium catenatum*	42.8164

MiD53-1	*M. itinerans*	TaD53-2	*Triticum aestivum*	27.3073
MiD53-1	*M. itinerans*	TaD53-1	*Triticum aestivum*	28.5442
MiD53-1	*M. itinerans*	TaD53-3	*Triticum aestivum*	24.1675
MiD53-1	*M. itinerans*	TaD53-4	*Triticum aestivum*	27.9734
MiD53-1	*M. itinerans*	TaD53-5	*Triticum aestivum*	23.882
MiD53-1	*M. itinerans*	TaD53L	*Triticum aestivum*	27.4025
MiD53-1	*M. itinerans*	TuD53L	*Triticum urartu*	23.882
MiD53-1	*M. itinerans*	TdD53-1	*Triticum dicoccoides*	28.5442
MiD53-1	*M. itinerans*	TdD53-2	*Triticum dicoccoides*	27.5928
MiD53-1	*M. itinerans*	TdD53-3	*Triticum dicoccoides*	23.882
MiD53-1	*M. itinerans*	TdD53-4	*Triticum dicoccoides*	27.9734
MiD53-1	*M. itinerans*	PhD53	*Panicum hallii*	31.0181
MiD53-1	*M. itinerans*	ZmD53	*Zea mays*	22.8354
MiD53-1	*M. itinerans*	SbD53-1	*Sorghum bicolor*	25.3092
MiD53-1	*M. itinerans*	SbD53-2	*Sorghum bicolor*	26.3559
MiD53-1	*M. itinerans*	OsD53	*Oryza sativa Japonica Group*	27.4976
MiD53-1	*M. itinerans*	OsD53L	*Oryza sativa Japonica Group*	28.0685
MiD53-1	*M. itinerans*	ObD53	*Oryza brachyantha*	27.8782
MiD53-1	*M. itinerans*	ObD53L	*Oryza brachyantha*	30.7326
MiD53-2	*M. itinerans*	LZmD53	*Zostera marina*	25.3998
MiD53-2	*M. itinerans*	PeD53L	*Phalaenopsis equestris*	36.7827
MiD53-2	*M. itinerans*	DcD53	*Dendrobium catenatum*	36.2183
MiD53-2	*M. itinerans*	TaD53-2	*Triticum aestivum*	29.445
MiD53-2	*M. itinerans*	TaD53-1	*Triticum aestivum*	25.1176
MiD53-2	*M. itinerans*	TaD53-3	*Triticum aestivum*	29.8213
MiD53-2	*M. itinerans*	TaD53-4	*Triticum aestivum*	26.8109
MiD53-2	*M. itinerans*	TaD53-5	*Triticum aestivum*	29.8213
MiD53-2	*M. itinerans*	TaD53L	*Triticum aestivum*	29.7272
MiD53-2	*M. itinerans*	TuD53L	*Triticum urartu*	29.9153
MiD53-2	*M. itinerans*	TdD53-1	*Triticum dicoccoides*	25.1176
MiD53-2	*M. itinerans*	TdD53-2	*Triticum dicoccoides*	29.7272
MiD53-2	*M. itinerans*	TdD53-3	*Triticum dicoccoides*	29.8213
MiD53-2	*M. itinerans*	TdD53-4	*Triticum dicoccoides*	26.7168
MiD53-2	*M. itinerans*	PhD53	*Panicum hallii*	31.5146
MiD53-2	*M. itinerans*	ZmD53	*Zea mays*	28.5983
MiD53-2	*M. itinerans*	SbD53-1	*Sorghum bicolor*	28.6924
MiD53-2	*M. itinerans*	SbD53-2	*Sorghum bicolor*	31.5146
MiD53-2	*M. itinerans*	OsD53	*Oryza sativa Japonica Group*	28.5042
MiD53-2	*M. itinerans*	OsD53L	*Oryza sativa Japonica Group*	33.4901
MiD53-2	*M. itinerans*	ObD53	*Oryza brachyantha*	30.2916
MiD53-2	*M. itinerans*	ObD53L	*Oryza brachyantha*	28.5042
